# Shear wave elastography as a quantitative biomarker of diabetic peripheral neuropathy: A systematic review and meta-analysis

**DOI:** 10.3389/fpubh.2022.915883

**Published:** 2022-07-22

**Authors:** Bingtian Dong, Guorong Lyu, Xiaocen Yang, Huaming Wang, Yongjian Chen

**Affiliations:** ^1^Department of Ultrasound, The Second Affiliated Hospital of Fujian Medical University, Quanzhou, China; ^2^Department of Clinical Medicine, Quanzhou Medical College, Quanzhou, China; ^3^Department of Ultrasound, Chenggong Hospital, Xiamen University, Xiamen, China

**Keywords:** biomarker, diabetic peripheral neuropathy, diagnosis, stiffness measurement, shear wave elastography

## Abstract

**Background:**

Diabetic peripheral neuropathy (DPN) is one of the most common chronic complications of diabetes and the strongest initiating risk factor for diabetic foot ulceration. Early diagnosis of DPN through screening measures is, therefore, of great importance for diabetic patients. Recently, shear wave elastography (SWE) has been used as a method that is complementary to neuroelectrophysiological examination in the diagnosis of DPN. We aimed to conduct a meta-analysis based on currently available data to evaluate the performance of tibial nerve stiffness on SWE for diagnosing DPN.

**Methods:**

Both PubMed, EMBASE, the Cochrane Library, and Web of Science were searched for studies that investigated the diagnostic performance of SWE for DPN up to March 1th, 2022. Three measures of diagnostic test performance, including the summary area under receiver operating characteristics curve (AUROC), the summary sensitivity and specificity, and the summary diagnostic odds ratios were used to assess the diagnostic accuracy of SWE. All included studies were published between 2017 and 2021.

**Results:**

Six eligible studies (with 170 DPN patients, 28 clinically defined DPN patients, 168 non-DPN patients, and 154 control participants) that evaluated tibial nerve stiffness were included for meta-analysis. The summary sensitivity and specificity of SWE for tibial nerve stiffness were 75% (95% confidence interval [CI]: 68–80%) and 86% (95% CI: 80–90%), respectively, and the summary AUROC was 0.84 (95% CI: 0.81–0.87), for diagnosing DPN. A subgroup analysis of five two-dimensional SWE studies revealed similar diagnostic performance, showing the summary sensitivity and specificity of 77% (95% CI: 69–83%) and 86% (95% CI: 79–91%), respectively, and a summary AUROC value of 0.86 (95% CI: 0.83–0.89).

**Conclusions:**

SWE is found to have good diagnostic accuracy for detecting DPN and has considerable potential as an important and noninvasive adjunctive tool in the management of patients with DPN.

## Introduction

Diabetes is one of the most common chronic diseases worldwide and has become an important public health problem recently (1). According to the data of International Diabetes Federation (IDF) (2), ~463 million adults around the world were suffering from diabetes in 2019, and the diabetic population is expected to reach 700 million people by 2045 (about 10% of the global population).

Diabetic peripheral neuropathy (DPN), the main type of diabetic neuropathy, is one of the most common and serious complication of diabetes and the strongest initiating risk factor for diabetic foot ulceration, occurring in about 50% of patients with diabetes (3, 4). This percentage is even higher, up to 60–90% in some areas (5, 6). DPN is the leading cause of lower-limb amputation and disabling neuropathic pain (7), which has a devastating effect on the quality of life and long-term survival of patients with diabetes and brings a heavy economic burden. It is worth noting that major amputations in patients with diabetes are associated with a low life expectancy, with a 5-year mortality ranging from 52 to 80% (8). Therefore, early diagnosis of the DPN in people with diabetes is of great importance for taking effective targeted measures, thereby preventing the development of foot ulcers and amputations.

However, in clinical settings, the assessment of DPN can be challenging and is mainly based on characteristic symptoms and signs (3, 9). At present, nerve conduction studies (NCS) is widely considered to be one of the gold standard methods for evaluating DPN (10). NCS is a quantifiable, objective, and sensitive method. Nevertheless, there are some limitations of this technique, such as invasiveness, time-consuming, high cost, and the need for qualified professionals to perform (9), which has largely restricted their practical applications. Notably, NCS is limited to evaluating large nerve fibers, while small nerve fibers are the first to be affected in DPN patients (9). So this technique does not assess early neuropathic changes (3). Moreover, NCS has usage difficultly for screening in large sample sizes (11). Therefore, there is a pressing need for portable, reliable, and valid tools to detect DPN.

Over the past few years, shear wave elastography (SWE) has gathered considerable attention. SWE is a non-invasive imaging technique that maps the elastic properties of tissues by assessing the velocity of shear wave propagation in the particular tissue (12). The shear wave speed is directly related to tissue stiffness (12). This modality offers a new type of high-quality ultrasound examination and has been widely applied in many organs such as the liver (13, 14), thyroid (15, 16), and the breast (17, 18). As an exciting and rapidly evolving adjunctive diagnostic tool to conventional ultrasound, SWE provides more quantitative information of tissue properties that used in the routine clinical evaluation of various traumatic and pathological conditions of the musculoskeletal system, which may contribute to diagnosis (19). Interestingly, a recent study in which SWE technique was used as a method that is complementary to neuroelectrophysiological examination in the diagnosis of DPN has been found that the stiffness of the affected nerves of diabetic patients with DPN was significantly greater than that of diabetic patients without DPN and healthy control individuals (20).

Although several studies have evaluated the diagnostic performance of SWE in detecting DPN, most have included a relatively small sample size (20–23). Furthermore, a consensus for the value of SWE that used as a biomarker in the diagnosis of DPN has not been reached. Generating an evidence-based summary of the SWE performance characteristics would be of high clinical importance for improved management of DPN in diabetic patients. Given this, in the present study, we aimed to conduct a meta-analysis based on currently available data to evaluate the diagnostic accuracy of SWE for the detection of DPN.

## Methods

### Literature search strategy

The Preferred Reporting Items for Systematic Reviews and Meta-Analyses (PRISMA) 2020 guidelines were followed for reporting this systemic review and meta-analysis (24). Both PubMed, EMBASE, the Cochrane Library, and Web of Science were systematically searched from inception to March 1th, 2022 using the following keywords: ((“diabetes”) OR (“diabetic peripheral neuropathy”) OR (DPN) OR (“diabetic foot”) OR (“diabetic foot ulcers”) OR (DFU) OR (“diabetic complications”)) AND ((elastography)). In addition, references of the identified articles were manually examined for other relevant publications. In our study, the references were managed using EndNote X9 software (Clarivate Analytics, Philadelphia, PA, United States).

### Inclusion and exclusion criteria

The original research articles were included in the present study if they conformed the following criteria: (1) the study examined the diagnostic performance of SWE for detecting DPN; (2) the SWE was included as an index test; (3) all the research patients were patients with diabetes; (4) the study enrolled at least 10 patients with diabetes, and; (5) at least one 2 × 2 table (i.e., true-positive, false-positive, false-negative, and true-negative) of test performance can be constructed using the data extracted from the study. Studies fulfilling any of the following criteria were excluded: (1) studies were not relevant to SWE diagnosis (e.g., studies that used only strain elastography); (2) reviews, guidelines, conference abstracts, and author comments; (3) animal studies; (4) data incomplete; (5) duplicate publications, and; (6) studies published in non-English or non-Science Citation Index (SCI) journals.

### Data extraction

Two investigators (B.T. Dong and X.C. Yang) read the articles, and checked the study eligibility and quality independently. In our meta-analysis, Microsoft Excel 2019 was used to pre-design the data extraction form. The patients' data including number of patients, age, sex, and body mass index (BMI) were collected from each included article. Moreover, the outcome indicators including cut-off values, sensitivity, specificity, positive predictive value (PPV), negative predictive value (NPV), and area under the receiver operating characteristic curve (AUROC) values were also extracted from the included studies.

As for the technical characteristics of SWE, the various aspects of this technique were assessed as follows: (1) vendors; (2) type of elastography; (3) probes; (4) target nerve; (5) the number of repeated measurements performed per patient; (6) the representative value of elasticity (mean or median); (7) number of readers; (8) blinding to the reference standard, and; (9) time interval between SWE and reference.

### Quality assessment

The quality of the included studies was assessed by the Quality Assessment of Diagnostic Accuracy Studies 2 (QUADAS-2) tool (25). The four steps of searching literatures, selecting studies, extracting data, and checking the study quality were separately performed by B.T. Dong and X.C. Yang in this meta-analysis. All discrepancies were resolved by consensus of these three authors (B.T. Dong, X.C. Yang, and G.R. Lyu).

### Data synthesis and analysis

Stata version 15.0 (STATA Corp., TX, USA) was selected to perform all statistical analyses. Review Manager (version 5.4.1; Cochrane Collaboration, https://training.cochrane.org/online-learning/core-software/revman/revman-5-download) software was used to assess the methodological quality of the included studies (26). First, we extracted the raw data from all the included studies, and then 2 × 2 tables were reconstructed for further analysis. In our meta-analysis, three measures of diagnostic test performance, including the summary AUROC, the summary diagnostic odds ratio (DOR), and the summary sensitivity and specificity, were used with the aim of examining the accuracy of SWE for diagnosing DPN. Positive likelihood ratio (LR) and negative LR were also calculated. For each summary statistic, we computed the 95% confidence intervals (CIs). In addition, we also conducted a subgroup analysis in order to evaluate the diagnostic performance of the typical type of SWE technique (two-dimensional SWE). Further, the summary receiver operating characteristic (SROC) curve was constructed using the data from the studies included in our meta-analysis to calculate the summary AUROC of SWE for detecting DPN.

### Assessment of heterogeneity and publication bias

The Spearman correlation coefficient was calculated to evaluate the threshold effect of the included studies. The existence of threshold heterogeneity was considered when the *P* < 0.05. To evaluate the non-threshold heterogeneity of included studies, the Cochran's *Q*-test and inconsistency index (*I*^2^) statistic was used. *I*^2^ value was calculated in our analysis, and then used to describe the amount of non-threshold heterogeneity. Using the Cochran's *Q*-test and *I*^2^ statistic, *P* < 0.05 indicated statistically significant heterogeneity; an *I*^2^ value >50% may be considered as substantial heterogeneity. Furthermore, the Deeks' funnel plot was used to assess the potential publication bias of the SWE studies with regard to their performance in detecting DPN, with a *P* < 0.05 suggested significant bias.

## Results

### Characteristics of the retrieved studies

**Figure 1:**
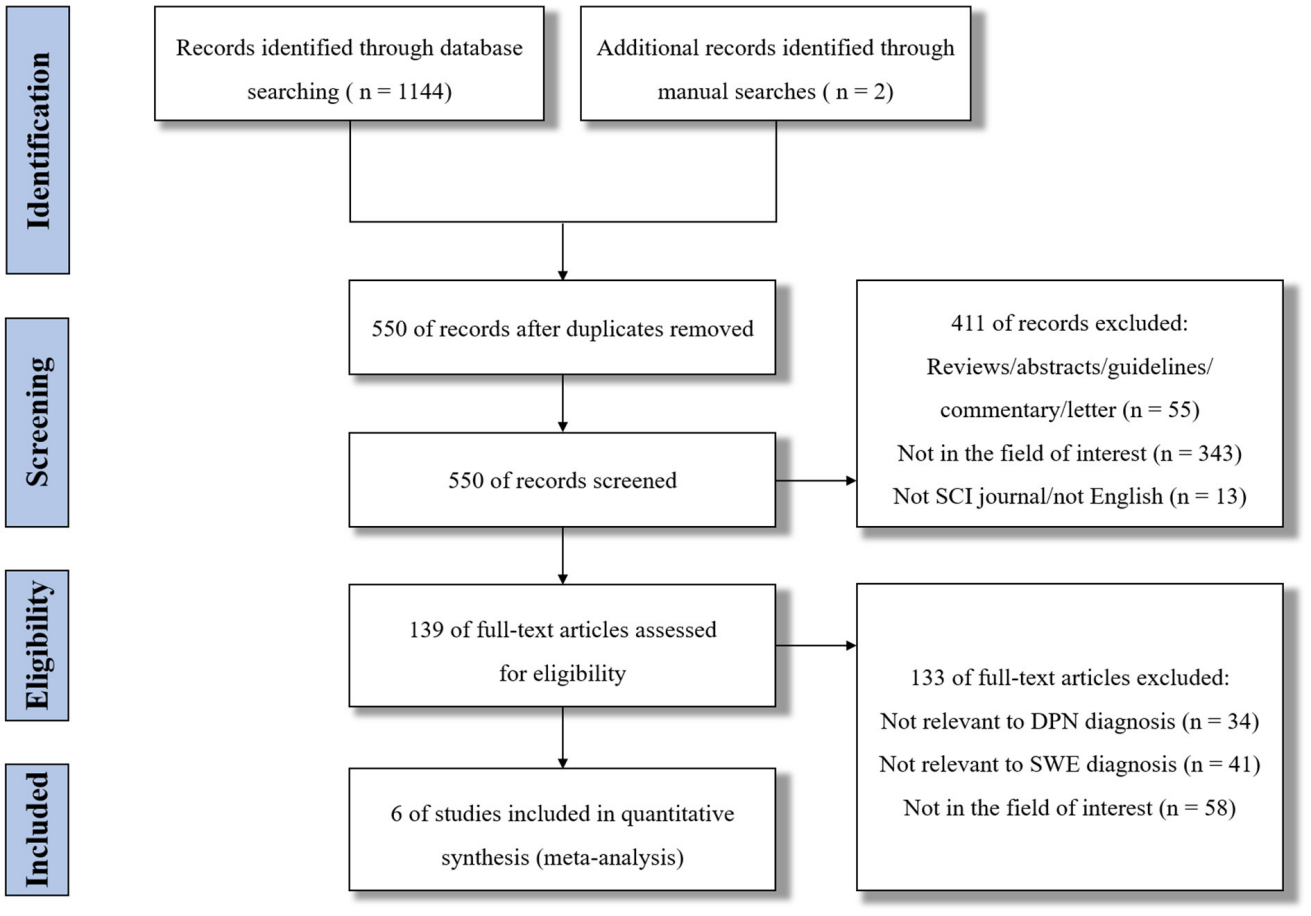
Flow diagram of study identification.

The flow diagram of study identification is shown in Figure 1. Using the search strategies presented, a total of 1,146 records were retrieved. After removal of 596 duplicates, 550 studies were initially screened. However, 544 studies were excluded for some reasons, such as reviews, only abstract, not relevant to DPN diagnosis, or not relevant to SWE diagnosis, etc. Finally, six studies were included for evaluation and meta-analysis (12, 20–23, 27).

Table 1 displays the basic characteristics of the studies included in this review. Most of the studies were published between 2019 and 2021, except one study that was published in 2017. One study cohort was from the Turkey, while the others were from the China. In addition, all six studies were conducted in a single-center setting. Among the included studies, there were 4 prospective studies. In terms of a reference standard used for diagnosis of DPN, three studies used the NCS, two used the electrophysiology examination, and one used the NCS and positive symptoms or signs of neuropathy. The articles included in this meta-analysis were published in six different journals, the mean impact factor of these journals was 4.638 (range: 1.889–11.105).

**Table 1:** Basic characteristics of the included studies.

**References, region**	**Study period**	**Center**	**Study design**	**Subject**	**Size**	**Median/mean age, years**	**BMI, kg/m** ^2^	**Male, %**
Dikici et al. (20), Turkey	Nov 2013–Jul 2014	One center	Prospective	DPN	20	60.0	31.4	50.0
				Non-DPN	20	61.0	29.8	40.0
				CG	20	58.0	28.7	45.0
Jiang et al. (21), China	Nov 2017–May 2018	One center	Prospective	DPN	25	66.2	24.3	44.0
				CDDPN^a^	25	60.9	24.2	24.0
				Non-DPN	20	57.1	25.4	40.0
				CG	20	57.8	24.2	50.0
He et al. (12), China	Nov 2016–Jul 2017	One center	NA	DPN	40	60.43	25.11	42.5
				Non-DPN	40	58.63	24.72	55.0
				CG	40	55.20	22.38	60.0
Wei et al. (22), China	Jun 2017–Sep 2017	One center	Prospective	Type 2 DM	30^b^	60.10	23.43	60.0
				CG	20	57.35	22.95	76.7
Chen et al. (23), China	Oct 2018–Aug 2019	One center	Prospective	DPN	30	54.43	25.67	60.0
				Non-DPN	33	54.85	26.19	45.5
				CG	33	51.51	23.28	42.4
Wang et al. (27), China	Dec 2017–Dec 2019	One center	NA	DPN	41	59.05	24.72	68.3
				Non-DPN	42	58.50	24.75	64.3
				CG	21	56.05	23.46	38.1

*BMI, body mass index; CDDPN, clinically defined diabetic peripheral neuropathy; CG, control group; DM, diabetes mellitus; DPN, diabetic peripheral neuropathy; NA, not available; Non-DPN, non-diabetic peripheral neuropathy*.

*^a^CDDPN, clinically defined DPN, which is defined as diabetic patients with clinical signs or symptoms of DPN but normal nerve conduction study (NCS)*.

*^b^The 30 patients with type 2 diabetes mellitus included 14 patients with a diagnosis DPN and 13 patients without a diagnosis of DPN, and 3 patients had positive symptoms or signs of neuropathy, but the NCS results were negative*.

### Characteristics of the study populations

**Figure 2:**
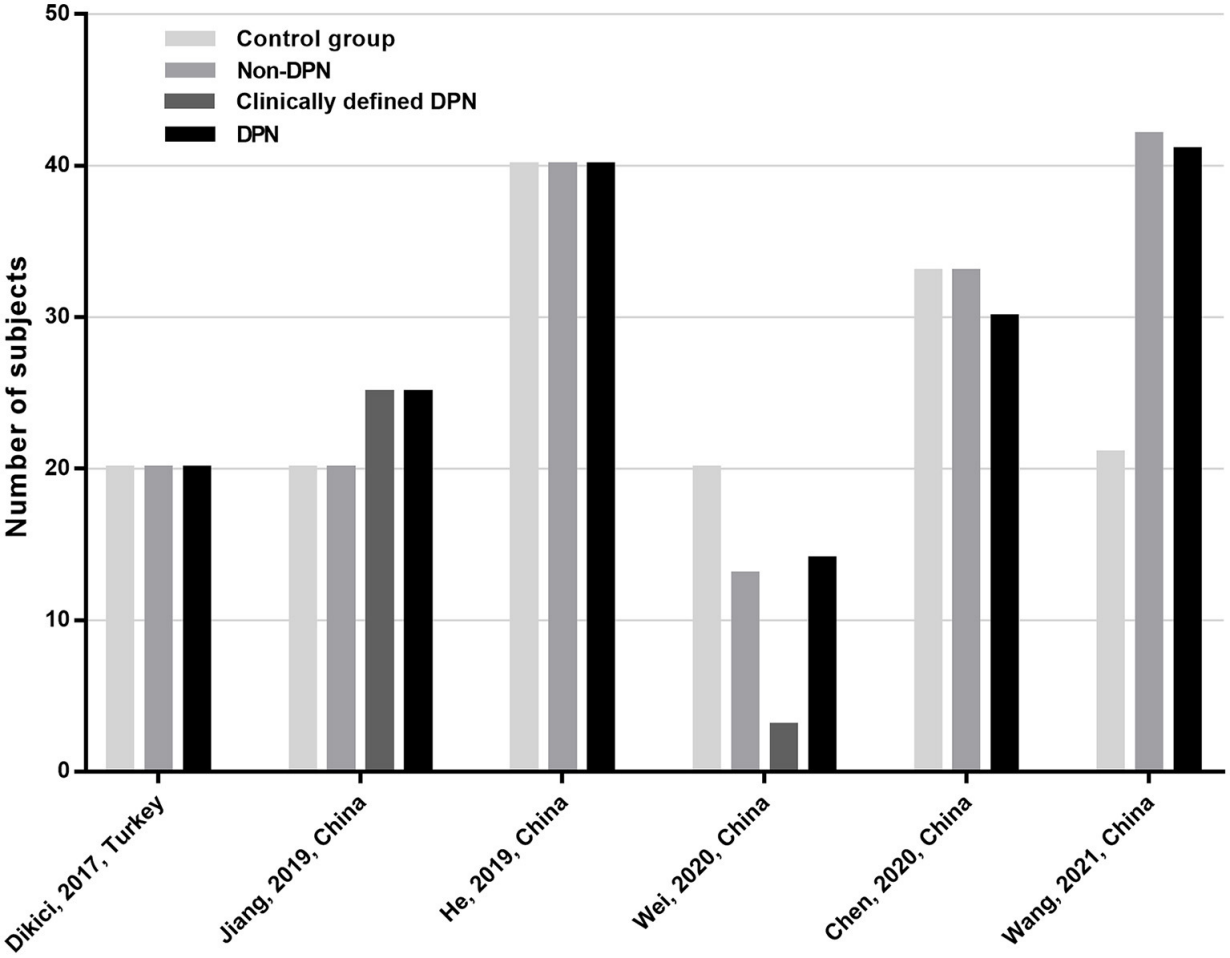
Distribution of research population of the included studies.

In total, 520 subjects were included in this meta-analysis. Specifically, they were 170 DPN patients, 28 clinically defined DPN patients (i.e., patients with clinical signs or symptoms of DPN but normal NCS), 168 non-DPN patients, and 154 control participants. In the DPN group, the study populations were all patients with type 2 diabetes mellitus (T2DM). Moreover, all the included subjects were adults. Figure 2 shows the distribution of research population of the included studies.

### Technical characteristics of shear wave elastography

**Figure 3:**
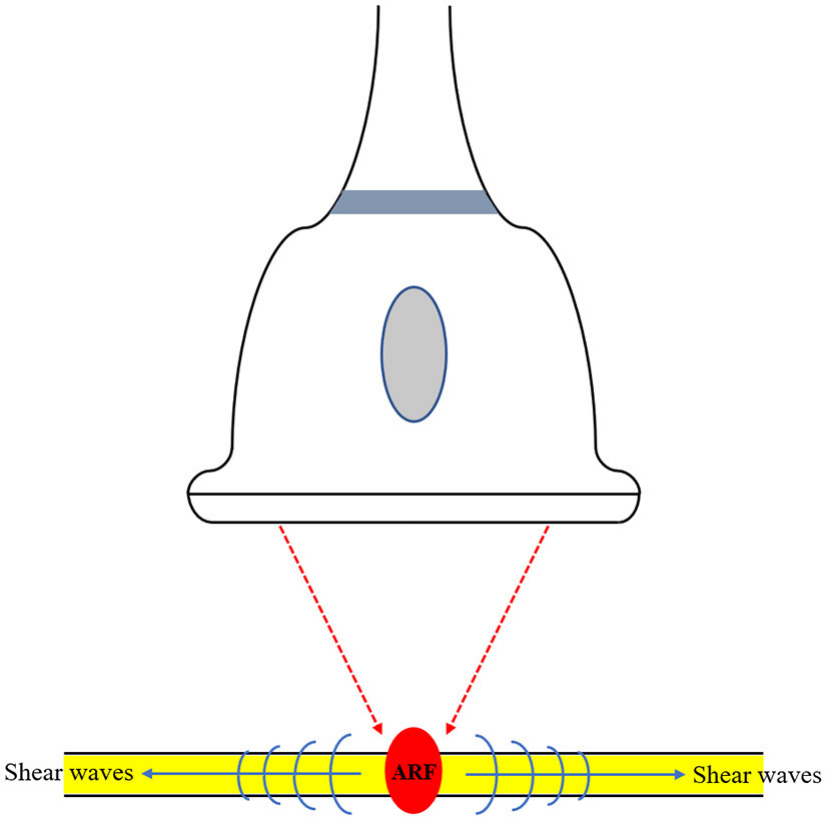
Illustration of the shear wave elastography (SWE) technique used in examination of nerves. SWE generates shear waves via acoustic radiation force (ARF). This technique provides more quantitative information in relation to the elasticity of the tissue.

The technical characteristics of the SWE technique used in the included studies are summarized in Table 2. Among the included studies, SWE was performed using two types of devices, including Aixplorer in five studies and Acuson S2000 in one study. Based on the technique used in this meta-analysis, SWE can be categorized as either point SWE, i.e., Virtual Touch Q (Siemens AG, Erlangen, Germany), or as two-dimensional SWE, i.e., Aixplorer (SuperSonic Imagine, Aix-en-Provence, France). The SWE technique used in examination of nerves is illustrated in Figure 3. Of note, all the 6 included studies measured tibial nerve stiffness, and two of these reports also included median nerve stiffness and common peroneal nerve stiffness measurements, respectively. Furthermore, as the measure of nerve stiffness, four two-dimensional SWE studies used elasticity, expressed in kilopascals (kPa), and one two-dimensional SWE study and one point SWE study used the shear wave speed, which expressed in meters per second.

**Table 2:** Technical characteristics of the elastography/reference standard used in the included studies.

**References, region**	**Technique**	**Elastography systems**	**Probe**	**No. of measurements**	**Representative values**	**Readers**	**Blinding**	**Time between Reference Standard and SWE**	**Reference standard**
Dikici et al. (20), Turkey	2D-SWE	Aixplorer^a^	4–15 MHz LAT	3	Mean	2	Yes	<1 week	NCS
Jiang et al. (21), China	2D-SWE	Aixplorer^a^	4–15 MHz LAT	4	Mean	2	Yes	NA	NCS
He et al. (12), China	2D-SWE	Aixplorer^a^	4–15 MHz LAT	3	Mean	2	Yes	NA	NCS
Wei et al. (22), China	p-SWE	Virtual Touch Q^b^	9L4 LAT	3	Mean	2	Yes	NA	NCS and positive symptoms or signs of neuropathy
Chen et al. (23), China	2D-SWE	Aixplorer^a^	4–15 MHz LAT	5	Mean	NA	NA	NA	Electrophysiology test
Wang et al. (27), China	2D-SWE	Aixplorer^a^	4–15 MHz LAT	3	Mean	1	Yes	<1 week	Electrophysiology test

*LAT, linear array transducer; NA, not available; NCS, nerve conduction study; p-SWE; point shear wave elastography; SWE, shear wave elastography; 2D-SWE, two-dimensional shear wave elastography*.

*^a^SuperSonic Imagine, Aix-en-Provence, France*.

*^b^Siemens AG, Erlangen, Germany*.

### Diagnostic accuracy

**Figure 4:**
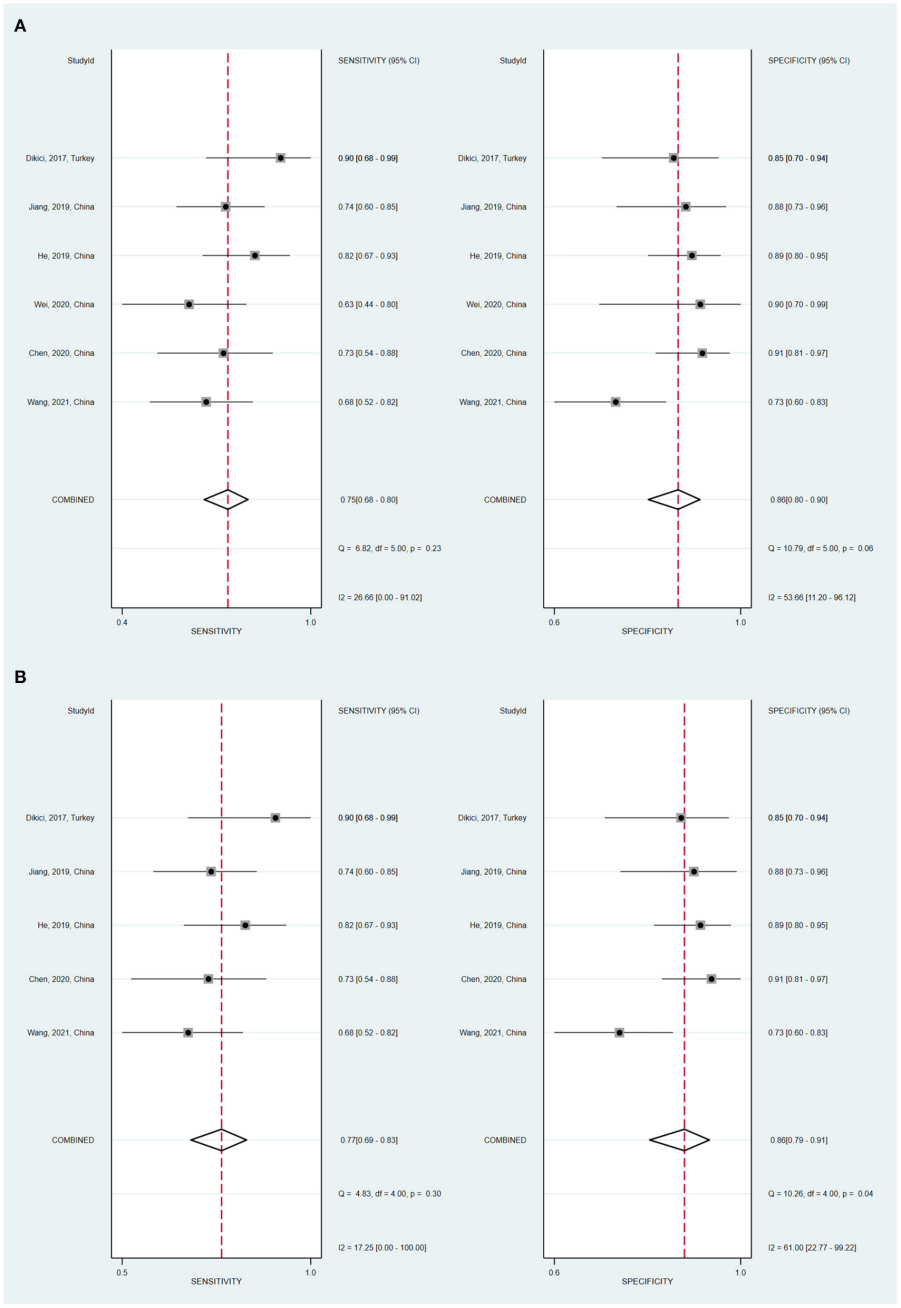
Coupled forest plots of the summary sensitivity and specificity of tibial nerve stiffness using shear wave elastography (SWE) **(A)** and two-dimensional SWE **(B)** for the diagnosis of diabetic peripheral neuropathy (DPN).

Six studies investigated SWE diagnostic performance for the prediction of DPN. As is shown in Table 3, in the 6 studies evaluating the tibial nerve stiffness using SWE for diagnosing DPN, the mean AUROC value was 0.864 (range: 0.712–0.941). Figure 4 demonstrates that, for the tibial nerve stiffness using SWE, the summary sensitivity and specificity were 75% (95% CI: 68–80%) and 86% (95% CI: 80–90%), respectively. For diagnosing DPN, the summary AUROC of SWE for tibial nerve stiffness was 0.84 (95% CI: 0.81–0.87), as shown in Table 4. The summary DOR was 18 (95% CI: 10–33), when tibial nerve stiffness was used to diagnose DPN. It is worth mentioning that tibial nerve stiffness measured by SWE had a sensitivity and specificity value of 90.0 and 85.0%, respectively, and an AUROC value of 0.941, at a cut-off value of 51.1 kPa, for predicting DPN.

**Table 3:** Summary of diagnostic accuracy of SWE for diagnosing DPN.

**Reference, region**	**Target nerve**	**Optimal EI outcome**	**Cut-off value**	**AUROC**	**Sensitivity, %**	**Specificity, %**	**PPV, %**	**NPV, %**
Dikici et al. (20), Turkey	TN	Mean	51.1 kPa	0.941	90.0	85.0	75.0	94.4
Jiang et al. (21), China	TN	Min	45.7 kPa	0.867	74.0	87.6	88.2	72.9
He et al. (12), China	TN	Mean	4.1 m/s	0.927	81.3	88.7	78.3	90.5
	MN	Mean	4.1 m/s	0.899	80.0	85.0	72.7	89.5
Wei et al. (22), China	TN	Mean	2.6 m/s	0.836	63.3	92.5	92.7	62.7
Chen et al. (23), China	TN	Mean	32.7 kPa	0.902	73.3	90.9	78.6	88.2
	CPN	Mean	NA	0.653	NA	NA	NA	NA
Wang et al. (27), China	TN	Mean	71.3 kPa	0.712	68.3	73.8	62.9	78.2

*AUROC, area under the receiver operating characteristic curve; CPN, common peroneal nerve; EI, elasticity indices; MN, median nerve; NA, not available; NPV, negative predictive value; PPV, positive predictive value; SWE, shear wave elastography; TN, tibial nerve*.

**Table 4:** Meta-analysis results of the stiffness of the tibial nerve using SWE for prediction of DPN.

	**No. of studies (Subjects)**	**Summary sensitivity (95% CI, %)**	**Summary specificity (95% CI, %)**	**Summary LR**+ **(95% CI)**	**Summary LR– (95% CI)**	**Summary AUROC (95% CI)**	**Summary DOR (95% CI)**
**Target nerve**							
Tibial nerve	6 (DPN, 170; CDDPN, 28; Non-DPN, 168; CG, 154)	75 (68–80)	86 (80–90)	5.3 (3.5–7.9)	0.30 (0.22–0.39)	0.84 (0.81–0.87)	18 (10–33)

*AUROC, area under the receiver operating characteristic curve; CDDPN, clinically defined diabetic peripheral neuropathy; CG, control group; CI, confidence interval; DOR, diagnostic odds ratio; DPN, diabetic peripheral neuropathy; LR+, positive likelihood ratio, LR–, negative likelihood ratio; SWE, shear wave elastography*.

**Figure 5:**
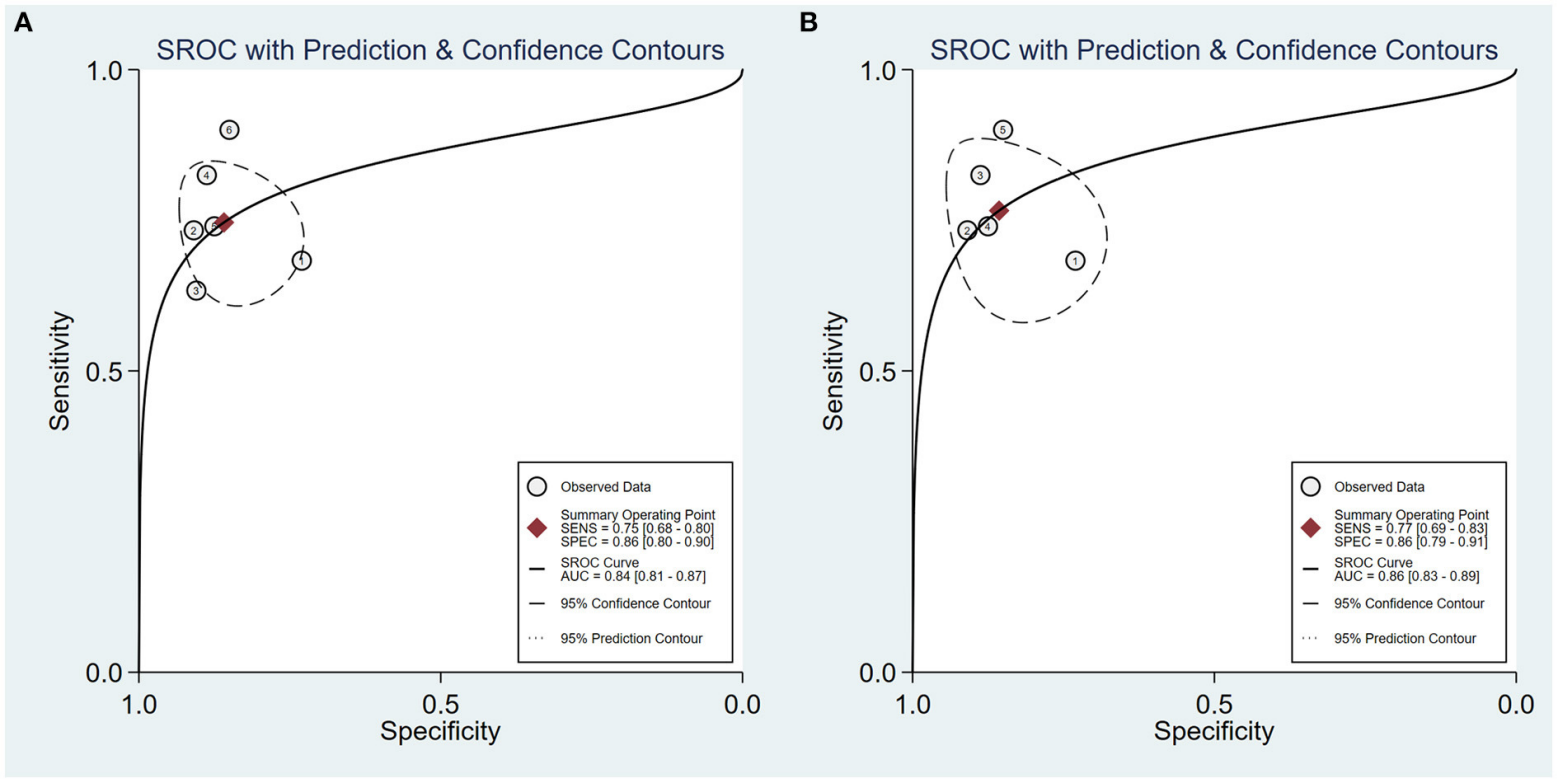
Summary receiver operating characteristic (SROC) curve of tibial nerve stiffness using shear wave elastography (SWE) **(A)** and two-dimensional SWE **(B)** for the diagnosis of diabetic peripheral neuropathy (DPN).

Furthermore, a subgroup analysis of five two-dimensional SWE studies revealed similar diagnostic performance, showing the summary sensitivity and specificity of 77% (95% CI: 69–83%) and 86% (95% CI: 79–91%), respectively, and a summary AUROC value of 0.86 (95% CI: 0.83–0.89) (Figure 5). Table 5 shows that the summary DOR of two-dimensional SWE for tibial nerve stiffness was 20 (95% CI: 10–39), for predicting DPN.

**Table 5:** Meta-analysis results of the stiffness of the tibial nerve using 2D-SWE for prediction of DPN.

	**No. of studies (Subjects)**	**Summary sensitivity (95% CI, %)**	**Summary specificity (95% CI, %)**	**Summary LR**+ **(95% CI)**	**Summary LR– (95% CI)**	**Summary AUROC (95% CI)**	**Summary DOR (95% CI)**
**Target nerve**							
Tibial nerve	5 (DPN, 156; CDDPN, 25; Non-DPN, 155; CG, 134)	77 (69–83)	86 (79–91)	5.3 (3.5–8.3)	0.27 (0.20–0.38)	0.86 (0.83–0.89)	20 (10–39)

*AUROC, area under the receiver operating characteristic curve; CDDPN, clinically defined diabetic peripheral neuropathy; CG, control group; CI, confidence interval; DOR, diagnostic odds ratio; DPN, diabetic peripheral neuropathy; LR+, positive likelihood ratio, LR–, negative likelihood ratio; SWE, shear wave elastography; 2D-SWE, two-dimensional shear wave elastography*.

Notably, there was one study in which SWE measurements were performed on both the median nerve and tibial nerve; this study simultaneously reported the diagnostic performance of median nerve stiffness (cut-off value, 4.1 m/s; sensitivity, 80.0%; specificity, 85.0%; AUROC, 0.899) and tibial nerve stiffness (cut-off value, 4.1 m/s; sensitivity, 81.3%; specificity, 88.7%; AUROC, 0.927). Additionally, another study also reported that common peroneal nerve measured by SWE had an AUROC of 0.653 for diagnosing DPN.

### Heterogeneity and publication bias

No threshold effect was found in the present meta-analysis, as depicted by the Spearman correlation coefficient value of 0.257 (*P* = 0.623). When SWE technique was used to diagnose DPN, the Cochran's Q-test (sensitivity, *P* = 0.23; specificity, *P* = 0.06) showed no statistically significant heterogeneity evidence both with regard to the summary sensitivity and specificity. This can be seen in Figure 4.

**Figure 6:**
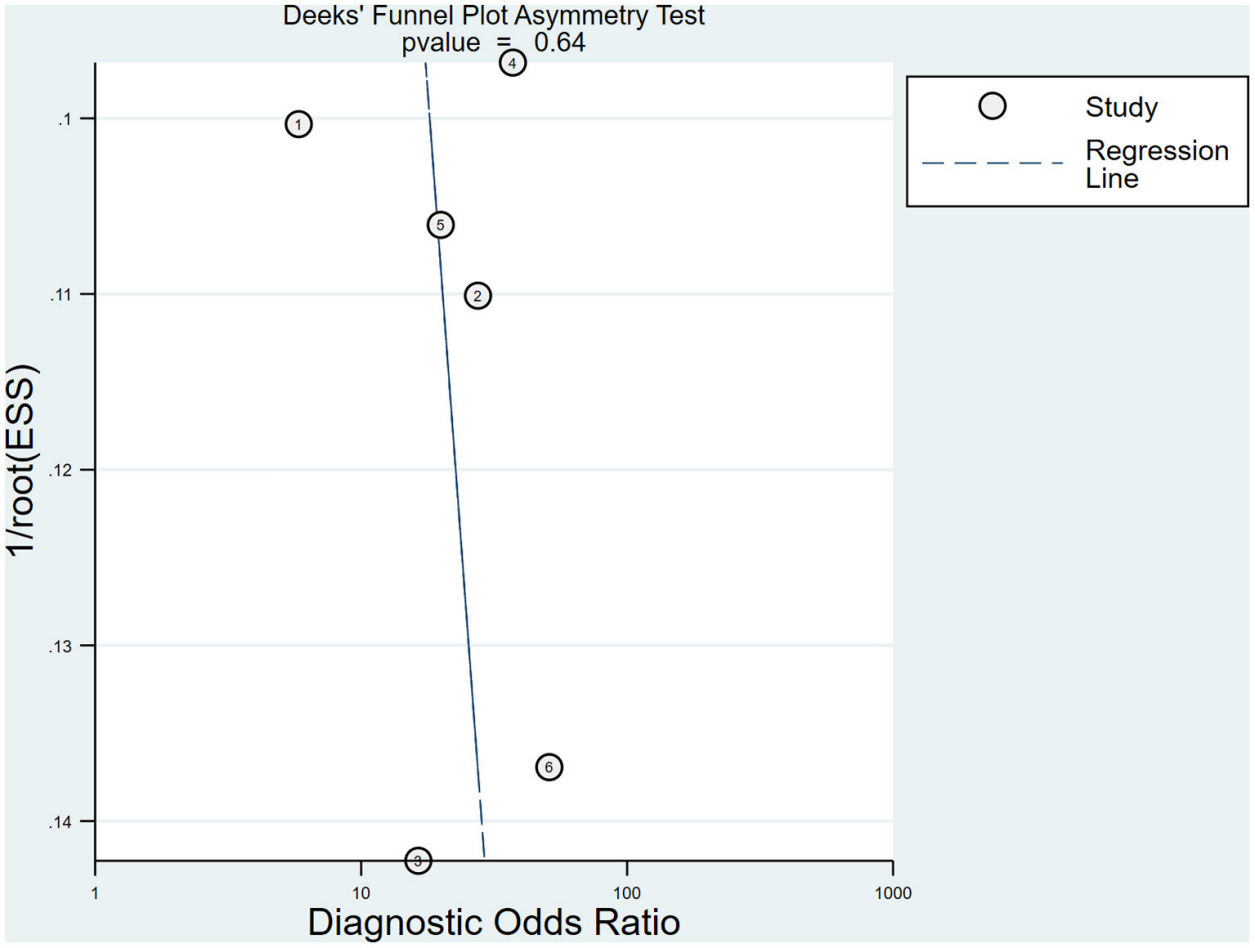
Deeks' funnel plot used to assess publication bias.

Deeks' funnel plot of SWE used to assess publication bias is illustrated in Figure 6. In this meta-analysis, there was no evidence of publication bias among the six included studies with a *P* value of 0.64.

## Discussion

DPN is the most important risk factor for the occurrence of diabetic foot ulcers, which seriously affect the quality of life and survival of patients with diabetes (3, 9). Early diagnosis of DPN is, therefore, of increasing importance. Unfortunately, in routine clinical practice, there are currently no simple biomarkers for early detection of DPN (3). In this regard, a novel diagnostic tool known as SWE technique may provide valuable alternatives, which has recently been introduced for the detection of DPN. In view of this, we wondered whether SWE can be used as a biomarker for the diagnosis of DPN. Our meta-analysis presents a comprehensive summary of the SWE performance characteristics for DPN, and, to our knowledge, this is the first meta-analysis of published studies that provides evidence of SWE being a novel and non-invasive tool for diagnosing DPN.

In this systematic review and meta-analysis, we identified six (170 DPN patients, 28 clinically defined DPN patients, 168 non-DPN patients, and 154 control participants) original articles with enough data to assess the performance of SWE for the prediction of DPN. Our study has revealed that tibial nerve stiffness measurement by SWE has good diagnostic performance for DPN, showing a summary sensitivity and specificity of 75 and 86%, respectively, and a summary AUROC of 0.84. A subgroup analysis of five two-dimensional SWE studies revealed similar diagnostic performance. The summary sensitivity and specificity were 77 and 86%, respectively, and the summary AUROC was 0.86. These results thus indicate that SWE can be used as a potential novel, useful, and quantitative tool for diagnosing DPN.

Tibial nerve is the most frequently involved site in diabetic polyneuropathy (20). Indeed, previous work has clearly shown that, when measured with SWE, the tibial nerve is stiffer in patients with diabetes. Several pathophysiological mechanisms may be used to explain changes in nerve stiffness as reflected by SWE measurements. The DPN developed because of the metabolic disorders associated with chronic hyperglycemia; edema within the nerve fascicle can increase intraneural pressure and then make the nerve stiffer (21, 27).

Of interest, even in diabetic patients without DPN, the stiffness of the tibial nerve was significantly higher than that of healthy control subjects (20). However, both neuroelectrophysiological examination and cross-sectional area (CSA) at ultrasound examination did not reflect such a change between diabetic patients without DPN and healthy control subjects (20). Notably, in the previous original study conducted by Jiang et al. (21), the results had shown that clinically defined DPN patients, i.e., patients with clinical signs or symptoms of DPN but normal NCS which often occurs in the early stages of DPN (28), had significantly greater tibial nerve stiffness than both diabetic patients without DPN and control subjects. These diabetic patients may have already suffered some nerve damage, although normal electrophysiological examination results exists (23). Which suggests that the SWE technique may be able to detect DPN before it becomes evident clinically or on NCS (29). This finding also shows that SWE, compared to electrophysiology test, exhibits a better correlation with clinical findings (21). Therefore, SWE technique has more potential value in early subelectrophysiological DPN detection. Early diagnosis of DPN is important, as early treatment at the earliest stages of DPN decreases both short-term and long-term morbidity (30, 31). Nevertheless, in view of the limited sample size, future studies using the SWE technique to assess the nerve stiffness in patients with clinically defined DPN are clearly needed.

In our meta-analysis, five of the six included studies used two-dimensional SWE technique for nerve stiffness assessment, and the other used point SWE technique. Both of these techniques rely on the acoustic radiation force impulse (ARFI) technique, which generates shear waves using focused, short-duration acoustic pulses (32). In contrast, two-dimensional SWE represents a relatively new ultrasound elastography technology for quantitative estimation of tissue stiffness (33). Two-dimensional SWE is a real-time and noninvasive imaging technique, which has distinct strengths for the evaluation of peripheral neuromuscular disorders. Surprisingly, one of the earliest studies using this technique showed that two-dimensional SWE displayed high sensitivity (90%) and specificity (85%) in the diagnosis of DPN, outperforming the CSA measurements (20). Thus, two-dimensional SWE has the considerable potential to be a promising non-invasive tool for diagnosing DPN. Considering the limited number of included studies, however, we were unable to compare these two SWE techniques in this meta-analysis. Additional studies are therefore needed to further investigate the performance differences between two-dimensional SWE and point SWE.

Notably, across the studies included in our meta-analysis, the cut-off values used to diagnose DPN varied. Several factors, such as imaging plane (longitudinal or axial) and the size of the region of interest while performing the elastography, limb position, different anatomic regions, and sometimes a variable distribution of the severity of diabetes, may have contributed to these differences. A previous study has examined the effect of limb position on the stiffness of the tibial nerve measurement by SWE technique (34). There may also be other factors that may simultaneously affect the cut-off value. In actual clinical practice, determining the optimal cut-off value for nerve SWE measurements is very important in order to ensure its general clinical applicability. Additionally, if used in combination with other methods such as the Toronto clinical scoring system (TCSS), SWE technique could potentially further improve the diagnostic value for DPN (27).

This meta-analysis has some limitations that should be noted. First, of the six included studies, five were from China. Therefore, the generalization of the present meta-analysis findings is relatively limited. Second, DPN is a multiple peripheral nerve disease; we only summarized the diagnostic value of SWE in the detection of DPN of the tibial nerve. It is worth stating that, in two of the six included studies (12, 23) that also reported the diagnostic performance of median nerve and common peroneal nerve, respectively, the results showed that the tibial nerve on SWE had better performance for diagnosing DPN. Third, our meta-analysis maybe have several intrinsic heterogeneities, such as techniques, reference standards, and SWE measurements. Furthermore, the optimal thresholds were not determined in this meta-analysis. Therefore, further studies with a larger sample size are needed. Finally, we only focused on the full-articles published with English, which may bias the results.

In conclusion, our meta-analysis demonstrated that SWE shows good performance in diagnosing DPN and has considerable potential as an important and noninvasive adjunctive tool in the management of patients with DPN. Further studies focusing on the identification of optimal cut-off value for nerve SWE measurements are required in order to ensure its general clinical applicability.

## Data availability statement

The original contributions presented in the study are included in the article/Supplementary Material, further inquiries can be directed to the corresponding authors.

## Author contributions

BD, YC, and GL contributed to the study design and literature search. XY, HW, and YC completed the data analysis. BD, HW, and YC generated and improved the figures and tables. BD completed the manuscript. BD and GL proofread the manuscript. All authors contributed to the article and approved the submitted version.

## Conflict of interest

The authors declare that the research was conducted in the absence of any commercial or financial relationships that could be construed as a potential conflict of interest.

## Publisher's note

All claims expressed in this article are solely those of the authors and do not necessarily represent those of their affiliated organizations, or those of the publisher, the editors and the reviewers. Any product that may be evaluated in this article, or claim that may be made by its manufacturer, is not guaranteed or endorsed by the publisher.
